# The etherase system of *Novosphingobium* sp. MBES04 functions as a sensor of lignin fragments through phenylpropanone production to induce specific transcriptional responses

**DOI:** 10.1111/1758-2229.13210

**Published:** 2023-11-10

**Authors:** Eri Kumagawa, Madoka Katsumata, Hiroshi Nishimura, Takashi Watanabe, Shun'ichi Ishii, Yukari Ohta

**Affiliations:** ^1^ Gunma University Center for Food Science and Wellness, Gunma University Maebashi Gunma Japan; ^2^ Research Institute for Sustainable Humanosphere Kyoto University Uji Kyoto Japan; ^3^ Institute for Extra‐cutting‐edge Science and Technology Avant‐garde Research (X‐star) Japan Agency for Marine‐Earth Science and Technology (JAMSTEC) Yokosuka Kanagawa Japan

## Abstract

The MBES04 strain of *Novosphingobium* accumulates phenylpropanone monomers as end‐products of the etherase system, which specifically and reductively cleaves the β‐O‐4 ether bond (a major bond in lignin molecules). However, it does not utilise phenylpropanone monomers as an energy source. Here, we studied the response to the lignin‐related perturbation to clarify the physiological significance of its etherase system. Transcriptome analysis revealed two gene clusters, each consisting of four tandemly linked genes, specifically induced by a lignin preparation extracted from hardwood (*Eucalyptus globulus*) and a β‐O‐4‐type lignin model biaryl compound, but not by vanillin. The most strongly induced gene was a 2,4′‐dihydroxyacetophenone dioxygenase‐like protein, which leads to energy production through oxidative degradation. The other cluster was related to multidrug resistance. The former cluster was transcriptionally regulated by a common promoter, where a phenylpropanone monomer acted as one of the effectors responsible for gene induction. These results indicate that the physiological significance of the etherase system of the strain lies in its function as a sensor for lignin fragments. This may be a survival strategy to detect nutrients and gain tolerance to recalcitrant toxic compounds, while the strain preferentially utilises easily degradable aromatic compounds with lower energy demands for catabolism.

## Graphical abstract

*Novosphingobium* sp. MBES04 possesses a unique ability to sense lignin fragments by producing phenylpropanones which induce specific transcriptional responses. The strongly induced clusters involved in energy production by oxidative degradation and multidrug resistance. This adaptation may be a survival strategy, whereby the strain MBES04 preferentially uses easily degradable aromatic compounds as energy, while acquiring tolerance to toxic compounds when in contact with lignin‐related compounds and enabling them to serve as energy sources.

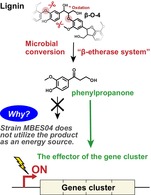

## INTRODUCTION

Lignin is a complex aromatic polymer and a major component of plant cell walls (Vanholme et al., 2010). In nature, peroxidases, oxidases and other accessory enzymes play major roles in breaking down lignin into small molecules (Abdel‐Hamid et al., 2013; Bugg et al., 2011; Martinez et al., 2005). These processes are mainly carried out by fungi, such as white rot fungi, whereas bacterial contributions are limited, especially in terms of the depolymerisation of high‐molecular‐weight lignin (Abdel‐Hamid et al., 2013). However, the metabolic capabilities of bacteria are versatile and crucial for the mineralisation of low‐molecular‐weight aromatic compounds, including partially degraded lignin fragments (Bugg et al., 2011).

The degradation pathways of lignin‐derived aromatic compounds in bacteria have been studied in various bacterial phyla, including Actinobacteriota, Proteobacteria (Psedomonadota) (Bugg et al., 2011; Silva et al., 2021), Bacillota (Khan et al., 2022; Mesle et al., 2022) and Bacteroidota (Taylor et al., 2012; Wu & He, 2013). Metagenomic approaches (Silva et al., 2021) have further expanded the known scope of bacterial metabolic capacities during lignin degradation. The mineralisation of low‐molecular‐weight aromatic compounds generally involves oxidative cleavage of aromatic rings followed by degradation in the β‐ketoadipate pathway, which is connected to the tricarboxylic acid cycle (Granja‐Travez et al., 2020). Therefore, such mineralisation processes are generally recognised as energy‐acquisition strategies (Granja‐Travez et al., 2020; Liu et al., 2019).

Among the aforementioned bacterial groups, most intensive studies have been conducted using the strain SYK‐6 affiliated with the genus *Sphingobium*. The enzymatic system that degrades the various lignin‐derived biaryl and monoaryl compounds is composed of various redox enzymes (Kamimura et al., 2017). A particularly distinctive feature of the strain SYK‐6 is that it has a cascade enzyme system, called the etherase system, which specifically catalyses the reductive cleavage of the β‐O‐4 ether bond, the major intramolecular bond of lignin. Etherase systems have been detected exclusively in bacteria belonging to the genera *Sphingobium*, *Novosphingobium* and *Altererythrobacter* (Kumagawa et al., 2023; Voss et al., 2020) within the order Sphingomonadales.

**FIGURE 1:**
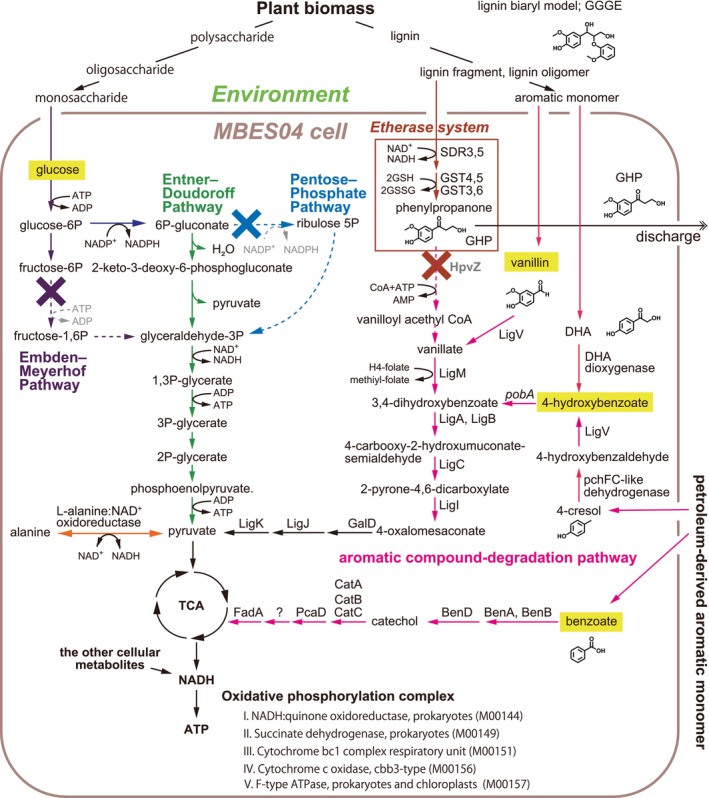
Proposed metabolic pathways of the MBES04 strain that are involved in glycolysis, the degradation of aromatic compounds and the etherase system. Embden–Meyerhof, Entner–Doudoroff and pentose phosphate pathways are shown in purple, green and blue, respectively. The etherase system and aromatics degradation pathways are shown in brown and magenta, respectively. The substrates that the strain MBES04 uses as a carbon source are indicated in yellow boxes (tested in a previous study, by Ohta et al., 2015). DHA, 2,4′‐dihydroxyacetophenone; GGGE, guaiacylglycerol‐β‐guaiacyl ether; GHP, guaiacylhydroxypropanone; GSH, reduced glutathione; GSSG, oxidised glutathione; GST, glutathione S‐transferase; SDR, short chain dehydrogenase reductase (Cα‐dehydrogenase).

In our earlier study, we isolated a *Novosphingobium* sp. strain MBES04 from deep‐sea sunken wood (Ohta et al., 2015). The genome of the strain MBES04 encodes enzymes involved in the etherase system (Figure 1), including Cα‐dehydrogenases (SDR3 and SDR5), etherases (GST4 and GST5) and glutathione lyases (GST3 and GST6) (Ohta et al., 2015). This combination of recombinant enzymes produced phenylpropanone monomers from the model β‐O‐4‐type lignin biaryl compounds, guaiacylglycerol‐β‐guaiacyl ether (GGGE) and lignin‐containing fractions extracted from wood (Ohta et al., 2017). When we cultured the strain in the presence of wood extract, we detected the accumulation of phenylpropanone monomers in the medium. However, the strain was unable to assimilate phenylpropanone monomers produced from the wood extract (Ohta et al., 2015). As the reductive cleavage reaction of the β‐O‐4 bond proceeds via net hydrogen transfer (Reiter et al., 2013), bacteria cannot gain energy from the reaction itself. The energy balance turns positive once the aromatic low‐molecular‐weight compounds are mineralised. These facts suggest that the etherase system of strain MBES04 is active and has a physiological significance other than energy acquisition from downstream metabolites.

In our previous study (Ohta et al., 2015), we performed gene expression analysis using MBES04 cells in the early logarithmic growth phase under supplementation of GGGE and its oxidised form, (2‐methoxyphenoxy) hydroxypropiovanillone (MPHPV), to identify candidate genes involved in the metabolism of lignin‐derived molecules. We observed weak induction of several enzymes that degrade aromatic monomers in response to GGGE supplementation. However, we did not observe activation of the etherase system or its downstream catabolic pathways of the key intermediate metabolites from partially degraded lignin, such as vanillin and protocatechuate. Furthermore, the addition of MPHPV appeared to suppress cellular energy production, including a decrease in cytochrome activity; however, the biological response to low‐molecular‐weight lignin and its physiological significance could not be elucidated.

The present study aimed to elucidate the physiological significance of the functional etherase system of strain MBES04, especially the reason why the products (phenylpropanone monomers) are not utilised as energy sources. We first reanalysed the genome sequence after circulation of the MBES04 genome to identify the full repertoire of genes responsible for aromatic‐compound metabolism and the energy‐acquisition pathways. Subsequently, the responses to lignin‐related aromatic small molecules were analysed using comparative transcriptome analysis. Furthermore, we investigated effector molecules to decipher associations between the etherase system and transcriptional responses to lignin fragments. Using these research approaches, we will understand how the bacterial species isolated from the wood sense lignin and acclimate to lignin‐existing environments, and we believe that such knowledge will be instrumental in the metabolic engineering for the production of valuable chemicals from lignin.

## EXPERIMENTAL PROCEDURES

### Chemicals

The chemicals used in this study were special grade and purchased from FUJIFILM Wako Pure Chemical Corporation (Osaka, Japan). Guaiacylglycerol‐β‐guaiacyl ether (GGGE), guaiacylhydroxylpropanone (GHP), syringylhydroxylpropanone (SHP) and veratryl alcohol (VA) were purchased from Tokyo Chemical Industry Co., Ltd. (Tokyo, Japan). Veratrylglycerol‐β‐guaiacyl ether (VGGE) and β‐guaiacyl‐α‐veratrylglycerone (GVG) were synthesised as previously described (Ohta et al., 2015). Luria‐Bertani (LB) medium, Miller was purchased from Nacalai (Kyoto, Japan). 2,4′‐dihydroxyacetophenone (DHA) was purchased from MolPort, Ltd. (Riga, Latvia). APA‐lignin was prepared from a wood meal of *Eucalyptus globulus* using acetic acid and peracetic acid mixture as a solvent under mild conditions at 50°C as described by Nishimura et al. (2022). Briefly, 1 g of Extractive‐free wood flour (*Eucalyptus globulus*) was milled in a ball mill (Planetary Ball Mill P‐6, Fritsch Japan Co. Ltd) for 3 h. This was subjected to microwave heating extraction using the acetic acid‐peracetcacid (APA) solution as the solvent. The extraction conditions are a microwave heating device (Initiator+, Biotage Japan Co., Ltd.) at 50°C for 10 min. The extract was concentrated by evaporating, followed by freeze‐drying to prepare APA‐lignin.

### Bacterial strains, culture media and growth conditions

The bacterial strains and plasmids used in this study are listed in Table 2. *Novosphingobium* sp. MBES04 (NBRC114556) (Ohta et al., 2015) was grown aerobically at 30°C in LB medium (Miller, 1972) supplemented with 5 mM MgSO_4_ as the basal medium. Strain MBES04 harbouring pQF‐*lacZ* derivative plasmid was cultured in the basal medium with tetracycline (10 μg mL^−1^ at the final concentration). In the experiments for the effects of carbon sources, strain MBES04 was grown in a mineral medium (Table S2) with the specified carbon sources. *E. coli* strains were grown at 37°C in an LB medium. When appropriate, antibiotics were added to the medium at the following final concentrations (μg mL^−1^): ampicillin 100; kanamycin 25; and streptomycin 50. Cell growth was followed by measuring turbidity at 600 nm.

### GHP production and growth in mineral medium with defined carbon sources

To examine the effect of carbon source addition on GHP production and growth, the medium was prepared by adding 20 mM glucose or 40 mM alanine to the mineral medium (Table S2). The strain MBES04 was grown in a basal medium for 16 h, harvested by centrifugation, and resuspended in a mineral medium with glucose or alanine or none (nutritional control), in the presence of 3 mM GGGE or DMSO (dissolution solvent of GGGE, solvent control). The OD600 was adjusted to 0.1 at inoculation. Thereafter, the strain was cultured at 30°C for 72 h and collected every 24 h for turbidity measurement at 600 nm and analysis using high‐performance liquid chromatography (HPLC), as described below.

### HPLC analysis

HPLC was performed using an Alliance 2796 (Waters) and a UV/Vis detector 2489 (Waters). The separation column was XBridgeC18 (particle size 3.5 μm, 4.6 × 100 mm: Waters) with 0.05% formic acid and 2 mM ammonium acetate added to ultrapure water as solvent A and methanol as solvent B. Gradient separation was performed at a flow rate of 1.2 mL/min (0–1 min, 10% methanol; 1–7 min, gradient from 10% to 100% methanol; and 7–10 min, 100% methanol). The detector was set at 270 nm and the column temperature at 40°C. Metabolite material samples were diluted 15‐fold in methanol and injected.

### DNA techniques and genome sequencing

Standard molecular biology techniques were carried out as previously described (Sambrook et al., 1989). Plasmid DNA was prepared with a Roche High Pure plasmid isolation kit (Merck KGaA, Darmstadt, Germany). DNA fragments were purified with a Wizard SV Gel and PCR Clean‐Up System (Promega, WI, USA). Oligonucleotide primers were supplied by FASMAC (Kanagawa, Japan) and their sequences are listed in Table S3. All cloned inserts and DNA fragments were confirmed by DNA sequencing through an Applied Biosystems 3730xl DNA Analyzer (Thermo Fisher Scientific, MA, USA). Strain MBES04 was grown aerobically overnight with shaking at 30°C in the basal medium. Cells were collected by centrifugation at 10,000 × *g* for 5 min at 4°C. Genomic DNA samples were purified using a NucleoSpin Plant II Midi kit (MACHEREY‐NAGEL GmbH & Co., Dueren, Germany). Genome sequencing was conducted using PacBio RS II and Illumina MiSeq and assembled using CLC De novo assembly v. 9. The updated complete genome sequences of strain MBES04 were deposited in GenBank/DDBJ/EMBL at accession numbers AP026899–AP026901. KEGG Automatic Annotation Server (KAAS) was used for the KEGG orthologous (KO) group assignment with the SBH (single‐directional best hit) method set to 45 as the threshold assignment score (Moriya et al., 2007).

### RNA purification

Strain MBES04 was grown aerobically for 12 h with shaking (160 rpm) at 30°C in LB medium supplemented with 5 mM MgSO_4_ (basal medium) to reach cell turbidity of 1.0 at OD600 and was then subcultured (approximately 1:20) to ensure cell turbidity of 0.05 at OD_600_ in 0.1 L of basal medium. After shaking (160 rpm) at 30°C for 12 h, cell turbidity was determined to be approximately 0.5. Cells in the mid‐log growth phase were collected by centrifugation and resuspended in a 1/10‐volume of basal medium, resulting in a 10‐fold concentrated cell suspension. The cells were then supplemented with vanillin (2.5 mM), GGGE (2.5 mM) and APA‐lignin (0.1 w/v%), with shaking at 30°C for 4 h. The 20‐fold stock solutions (100 mM vanillin and GGGE, 2.0 w/v% APA‐lignin) of the additives were prepared in DMSO. Cells cultured in basal medium with 5 v/v% DMSO without vanillin, GGGE or APA‐lignin were used as controls. Cells were collected by centrifugation at 10,000 × *g* for 5 min at 4°C. RNA was isolated and purified from the pelleted cells using the RNeasy plant min kit (Qiagen, Hilden, Germany) following the manufacturer's manual. Total RNA was eluted in 100 μL RNase‐free H_2_O and DNase I digestion of genomic DNA was then performed on a column using RNase‐free DNase I (Qiagen) according to the manufacturer's protocol. The obtained sample was then subjected to a second RNeasy purification step. RNA quality in the purified solutions was verified by electrophoresis on an Agilent Bioanalyzer to detect intact 16 S and 23 S rRNAs.

### Transcriptomic analysis

RNA sequencing libraries were constructed using DNA‐ and rRNA‐free RNA samples and were then sequenced using an Illumina Hiseq 2500 platform at the Macrogen Japan (Tokyo, Japan) following manufacturer's instructions (Seq 2500 System User Guide Document # 15035786 v01) using Sequencing Control Software (HCS 2.2.70). The RNA raw read data were deposited to the DDBJ/EMBL/GenBank databases under accession numbers DRR487002–DRR487005. The demultiplexed raw RNA sequence reads were quality‐trimmed by the Trim Read function with a quality score limit of 0.02 and an automatic adaptor trimming option on CLC Genomics Workbench version 20.0 (QIAGEN, Venlo, Netherlands). The obtained reads were separately mapped to strain MBES04 genome using the RNA‐Seq analysis function of CLC Genomics Workbench version 20.0 at the default parameters except for the length fraction of 1.0 and the similarity fraction of 1.0. The RPKM (Reads Per Kilobase per Million mapped reads) values (Mortazavi et al., 2008) were calculated to analyse the gene expression levels of each coding sequences (CDSs) of the strain MBES04 under different experimental conditions.

### Comparative genome analysis

Nucleotide sequences of main chromosomes for comparative genomics were retrieved from GenBank using the following accession numbers: FR856862.1 (*Novosphingobium* sp. PP1Y), CP000248.1 (*N. aromaticvorans* DSM12444), AP012222.1(*Sphingobium* sp. SYK‐6) and AP018498.1 (*Altereythrobacter* sp. B11). The dDDH values were calculated using the genome‐to‐genome distance calculator 3.0 and the built‐in programme gbdp2_blastplus (https://ggdc.dsmz.de/ggdc.php#). The orthoANI values were calculated using the calculation tool (version 0.93.1) by Lee et al. (2016).

### Cultivation of strain MBES04 in GGGE, vanillin, APA‐lignin

Overnight cultures of MBES04 cells in the basal medium were prepared. These cells were harvested and resuspended in the one‐tenth strength of LB medium with 5 mM MgSO_4_. The pre‐cultured cells were inoculated into one‐tenth strength LB medium with GGGE (2.5 mM), vanillin (2.5 mM), APA‐lignin (0.1%) and 5 mM MgSO_4_ to the OD600 of 0.1. DMSO (5.0%) was similarly added and used as control. The cultures were incubated with shaking at 30°C for 24 h. The culture samples at 0 and 24 h were immediately serially diluted with 1% NaCl containing 5 mM MgSO and inoculated with 50 μL on LB agar medium containing 5 mM MgSO_4_ and 50 μg mL^−1^ streptomycin. After incubation at 30°C for 48 h, the numbers of detectable colonies per mL unit were counted by visual observation and defined as CFU/mL. The difference of viable cell numbers between 0 and 24 h of cultivation was expressed as the increased shift of CFU by subtracting CFU_0 h_ from CFU_24 h_.

### Analysis of the reaction products from GGGE, vanillin, APA‐lignin by MBES04 cells

MBES04 cells were pre‐cultured overnight in a basal medium. The pre‐cultured cells were inoculated into basal medium containing GGGE (2.5 mM), vanillin (2.5 mM) and APA‐lignin (0.1%) to an OD600 of 0.1. The culture medium was shaken at 30°C for 24 h, and samples were collected every 2 h. The collected culture samples were supplemented with ethyl ferulate as an internal standard at a final concentration of 0.1 mM. Extraction was performed using triple volumes of ethyl acetate three times. The organic phase was collected and evaporated, and the residue was dissolved in 20% acetonitrile containing 0.1% formic acid for LC–MS analysis. LC–MS analysis was conducted under conditions according to the previous report by Ohta et al. (2017).

### Construction of promoter assay plasmid

The promoter assay plasmid with β‐galactosidase as a reporter (Figure S2A) was constructed using pQF‐*lacZ* as the parent plasmid (Table 2; Kaczmarczyk et al., 2013). The *lacZ* expression regulatory region of pQF‐*lacZ* was replaced by the putative promoter of cluster G‐II (designated as P_ClusterG‐II_). More specifically, P_ClusterG‐II_ (1–600 bp upstream region from start codon of MBENS4_1161 gene; Figure S2B) was amplified by PCR using the MBES04 genome as a temperate and a primer set of PClusterG‐II_Cloning_Fw and Rv (Table S3). The amplified fragment was cloned into the *lacZ*‐promoter and regulator less pQF‐*lacZ*, which was linearized by PCR using the primer set of Promoter infusion_Fw and Rv (Table S3) before the infusion cloning reaction (Takara Bio, Shiga, Japan). Maps of the constructed plasmid (pQF‐*lacZ*:: P_ClusterG‐II_) and nucleotide sequence of P_ClusterG‐II_ are shown in Figure S2.

### Assay for β‐galactosidase activity

The pQF‐*lacZ*:: P_ClusterG‐II_ was transformed into *E. coli* S17‐1 λpir and the transformant and strain MBES04 were co‐cultured for conjugative transmission. The promoter assay strain transformed by pQF‐*lacZ*:: P_ClusterG‐II_ was obtained by antibiotic selection and designated as strain MBE*lacZ*. The strain MBE*lacZ* was incubated for 16 h in basal medium containing 1 mM of the lignin‐related low molecular weight compounds (GGGE, MPHPV, VGGE, GVG, GHP, DHA, VA and 2,6‐dimethoxyphenol [2,6‐DMP]). The β‐galactosidase activities of the cultures were measured using *o*‐nitrophenyl β‐D‐galactopyranoside (ONPG) according to the method of Zhang and Bremer (1995), which was modified from the original method (Miller, 1972). In time‐course experiments, aliquots of independent quadruplicate cultures were sampled every 2 h. β‐Galactosidase activity was measured immediately after sampling. β‐Galactosidase activity was expressed in Miller Units (1000 × the mean rate of change in absorbance at 420 nm min^−1^ mL^−1^ per OD600 unit of culture). The error bars represent the standard error of the mean of quadruplicate experiments.

## RESULTS AND DISCUSSION

### Gene sets important for energy acquisition

**FIGURE 2:**
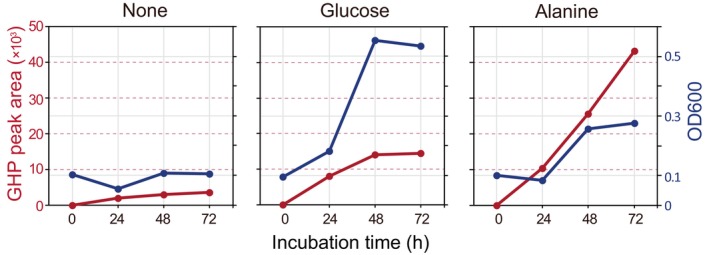
GHP production and growth of strain MBES04 in the presence of glucose or alanine. GHP production (red) from GGGE in the culture medium and growth of the strain (dark blue) in the mineral medium in the presence of glucose (20 mM) or alanine (40 mM) were monitored using HPLC quantification and optical density of the culture at 600 nm, respectively. Sampling was performed every 24 h for 72 h, including the time of inoculation. Abbreviations are listed in Figure 1.

Previously, we found that strain MBES04 possesses an active etherase system (Ohta et al., 2015), producing phenylpropane monomers from lignin model biaryls linked by β‐O‐4 ether bonds, the major bond in lignin molecules (GGGE and MPHPV), and lignin fragments (Figure 1). However, the strain was unable to assimilate the phenylpropanone monomer and discharged outside the cell (Ohta et al., 2015). In this study, we found that the addition of glucose or alanine to nutritionally limited media enhanced the production of GHP from GGGE during the growth of strain MBES04 (Figure 2). Considering that the etherase system consumes reducing power from 2 mol of reduced glutathione for every 1 mol of phenylpropanone monomer production (Figure 1), this finding indicates that an energy supply from other carbon sources helped activate the etherase system. To interpret the phenomena, we performed additional sequencing to fill the gap in the chromosome and searched for gene sets associated with pathways that acquire energy from other carbon sources.

Among the glycolytic pathways, the Embden–Meyerhof pathway (EMP) was defective because the strain lacks 6‐phosphofructokinase [EC: 2.7.1.11]. In addition, the strain lacks 6‐phosphogluconate dehydrogenase [EC: 1.1.1.44, EC: 1.1.1.343], which is required for the pentose phosphate pathway (PPP) (Spaans et al., 2015). In contrast, all enzymes required for the Entner–Doudoroff pathway (EDP) were present, suggesting that the EDP plays a central role in glucose metabolism in the strain MBES04. The strain possessed a complete set of genes that encode all five complexes required for oxidative phosphorylation. Thus, oxidative phosphorylation is important for energy acquisition in the strain.

The MBES04 genome validated the lack of orthologs of *hpvZ* that was previously identified as an essential enzyme for GHP (synonym for β‐hydroxypropiovanillone) catabolism in strain SYK‐6 (Higuchi et al., 2018). Higuchi et al. (2018) reported that the degradation of the propanone (C3) side chain of GHP proceeded via a coenzyme A (CoA)‐ and ATP‐demanding reaction. In contrast, we found that strain MBES04 has a gene repertoire enabling the complete catabolism of lignin‐derived aromatic monomers with a C1 side chain, such as vanillin and 4‐hydroxybenzoate (Figure 1), consistent with the results of previous culture experiments obtained using a single carbon source (Ohta et al., 2015). Strain MBES04 utilises benzoate as a sole carbon source (Ohta et al., 2015); however, 3‐oxoadipate CoA‐transferase (PcaIJ) orthologs were not identified in benzoate and catechol ortho‐cleavage pathways, suggesting the presence of alternative genes to PcaIJ. Considering the gene repertory and capability for carbon utilisation, the complex metabolic pattern of MBES04 enhances its robustness in terms of energy acquisition.

To compare the energy acquisition pathways with those of other strains in the family Sphingomonadaceae that possess etherase systems, we studied the *Novosphingobium* sp. PP1Y, *N. aromaticvorans* DSM12444, *Sphingobium* sp.SYK‐6 and *Altereythrobacter* sp. B11. Whole‐genome sequence similarities between the five strains were estimated using digital DNA–DNA hybridization (dDDH) (Meier‐Kolthoff et al., 2013) and improved versions of average nucleotide identity (OrthoANI) (Lee et al., 2016; Yoon et al., 2017) analyses. The dDDH and OrthoANI values of strain MBES04 versus the four strains ranged from 13.4 to 18.9 and from 70.3% to 76.6%, respectively (Table 1). The results showed that the genomic similarities amongthe strains are below the cut‐off for the species boundary (dDDH value >70%; OrthoANIu value >95%).

**TABLE 1:** Values of digital DNA–DNA hybridization (dDDH) and improved versions of average nucleotide identity (OrthoANI) using *Novosphingobium* sp. MBES04 as control.

Strain	*Novosphingobium* sp. PP1Y	*Novosphingobium aromaticvorans* DSM12444	*Sphingobium* sp. SYK‐6	*Altereythrobacter* sp. B11
orthoANI (%)	76.5	73.3	70.3	72.1
dDDH (%)	18.9	14.9	13.4	13.7

**TABLE 2:** Strain or plasmid used in this study.

Strain or plasmid	Relevant genotype and main characteristics	Reference or source
Strain		
*Novosphingobium* sp. strain MBES04	Wild‐type strain (NBRC114556)	Ohta et al. (2015)
MBE*lacZ*	MBES04 transformed by pQF‐*lacZ*:: P_ClusterG‐II,_ *Tcr*	This study
*Escherichia coli* DH5*α*	*F′, Φ80dlacZΔM15, Δ(lacZYA* ^*−*^ *argF)U169, deoR, recA1, endA1, hsdR17(rK* ^*−*^ *, mK* ^*+*^ *), phoA, supE44, λ* ^*−*^ *, thi‐1, gyrA96, relA1*	TOYOBO Co., LTD (Osaka, Japan)
*Escherichia coli* S17‐1 λpir	Tp^r^ Sm^r^ *recA thi hsdRM* ^+^ *RP42::.Tc::Mu::Km Tn7 λpir* phage lysogen	de Lorenzo and Timmis (1994)
Plasmids		
pQF	pCM62 with cymR*, PQ5 and MCS for N‐ and C‐terminal fusions to 3 × FLAG tag; Tcr (Addgene plasmid #48095)	Kaczmarczyk et al. (2013)
pQF‐*lacZ*	pQF with *lacZ* (Addgene plasmid #48094)	Kaczmarczyk et al. (2013)
pQF‐*lacZ*:: P_ClusterG‐II_	pQF‐*lacZ* with the 600 bp PCR amplicon carrying cluster G‐II promoter region (the map and sequence are shown in Figure S2)	This study

**FIGURE 3:**
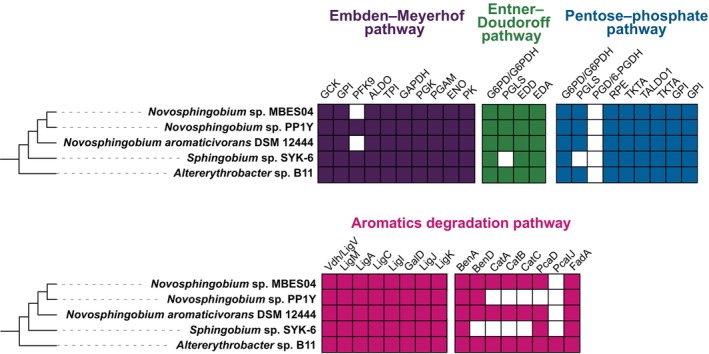
Comparison of the genome strain MBES04 with that of related strains possessing an etherase system. The phylogenetic tree of the strains possessing etherase systems was constructed using the neighbour‐joining method with a ribosomal protein gene (*rpsC*). The tiles show presence/absence of genes involved in glycolysis (Embden–Meyerhof pathway [purple], Entner–Doudoroff pathway [green] and pentose phosphate pathway [blue]), and aromatics degradation pathways (magenta) for vanillin (left panel) and benzoate/catechol (right panel). Annotation was primarily based on the classification of genes into Kyoto Encyclopedia of Genes and Genomes orthologous groups. Vanillin dehydrogenase was considered present if LigV orthologs (≥70% identity) were found using a BLASTP search. Abbreviations of the referenced enzymes are listed in Table S4.

Strains PP1Y, SYK‐6 and B11 shared defects in the PPP with strain MBES04. Strain DSM12444 shared defects in the EMP. As shown in Figure 3, with respect to glycolysis, all strains lacked at least one of the three pathways. Among Sphingomonadaceae strains possessing the etherase system, strain SYK‐6 was unable to grow sufficiently on most sugars and organic acids (Kamimura et al., 2017; Masai et al., 2007), which was caused by a lack of genes involved in the phosphotransferase system, resulting in insufficient sugar transport and phosphorylation (Varman et al., 2016).

Many gram‐negative bacteria preferentially use the EDP for glycolysis owing to its lower cost in terms of protein production and more favourable thermodynamic characteristics (Flamholz et al., 2013). The EDP efficiently yields NADPH, which confers a high tolerance to oxidative stress (Chavarria et al., 2013). In general, gram‐negative bacteria can obtain the energy necessary for survival via oxidative phosphorylation (Stettner & Segr é, 2013).

Recently, Linz et al. (2022) constructed a genome‐scale metabolic model of *N*. *aromaticivorans* to estimate the efficiency with which aromatic lignin was converted into valuable chemicals. The findings of their investigation suggest that energy derived from non‐aromatic carbon sources is crucial for the demethylation of lignin‐derived aromatic compounds. This is because the demethylation of methoxylated lignin‐derived aromatic compounds is not only an essential step (Abe et al., 2005) but also a rate‐limiting step that requires energy input (Venkatesagowda & Dekker, 2021) prior to energy production via β‐oxidation, which generates energy in the form of ATP after ring opening. Thus, diverse catabolic pathways for both aromatic and non‐aromatic compounds can compensate for the inefficient energy production from sugar metabolism.

### Gene expression in the presence of GGGE

To clarify the physiological significance of the β‐etherase system of strain MBES04, other than energy acquisition from downstream metabolites, transcriptomic analysis was conducted in the presence of GGGE. GGGE was used as a lignin‐mimicking small substrate cleaved by the β‐etherase system. Here, resting cells, which were prepared from cells aerobically cultured in nutritionally rich media at the growth phase, were used for RNA preparation to obtain sufficient biomass. These experimental settings were designed to avoid the adaptive responses for growth unrelated to β‐etherase reactions.

**FIGURE 4:**
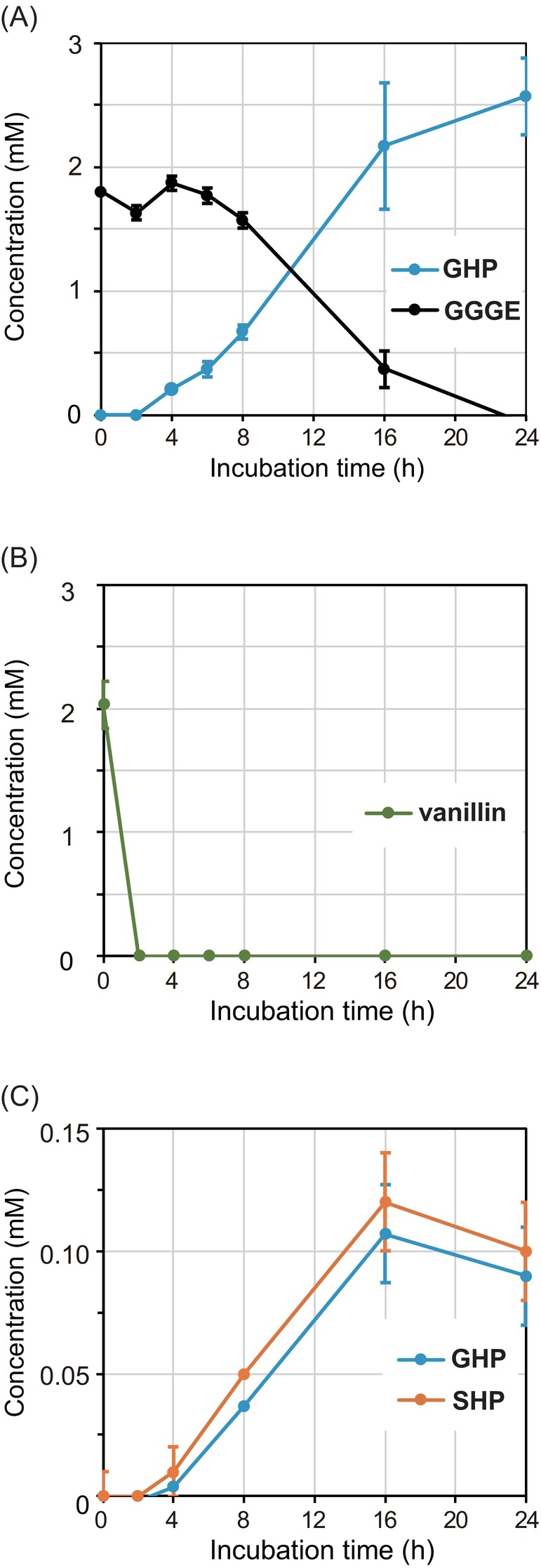
Analysis of reaction of MBES04 cells with GGGE, vanillin and APA. Overnight‐cultured MBES04 cells were collected and incubated in the basal medium with 2.5 mM GGGE (A), 2.5 mM vanillin (B), or 0.1% APA‐lignin (C) for 24 h at 30°C. The aliquots of reaction mixtures were extracted with ethyl acetate triplicate and analysed by LC–MS. For the quantification of the substrate, a calibration curve with standards was used, and extraction errors were adjusted by internal standards. The error bars represent the standard error of the mean of triplicate experiments.

To verify the viability of strain MBES04 cells in the presence of the substrates used in the subsequent transcriptomic experiments, the differences in the colony‐forming units (CFUs) between 0 and 24 h of cultivation were assessed in the presence or absence of the substrates in the nutritionally limited medium (one‐tenth strength of Luria‐Bertani (LB) medium containing 5 mM MgSO_4_) (Figure 4). The increase in CFU after inoculation was 2.6 ± 0.4 × 10^8^ CFU/mL in the presence of 2.5 mM GGGE, whereas 2.4 ± 0.1 × 10^8^ CFU/mL in the absence of GGGE (control). The growth of strain occurred at the same level in both conditions. Additionally, the reaction of 2.5 mM GGGE by the cell suspension was analysed using LC–MS (Figure 4A). At 4 h after exposure to GGGE, the production of GHP from GGGE reached a detectable level and was completed within 24 h, indicating the duration of the active metabolic capacity of the cells for subsequent transcriptomic experiments.

**FIGURE 5:**
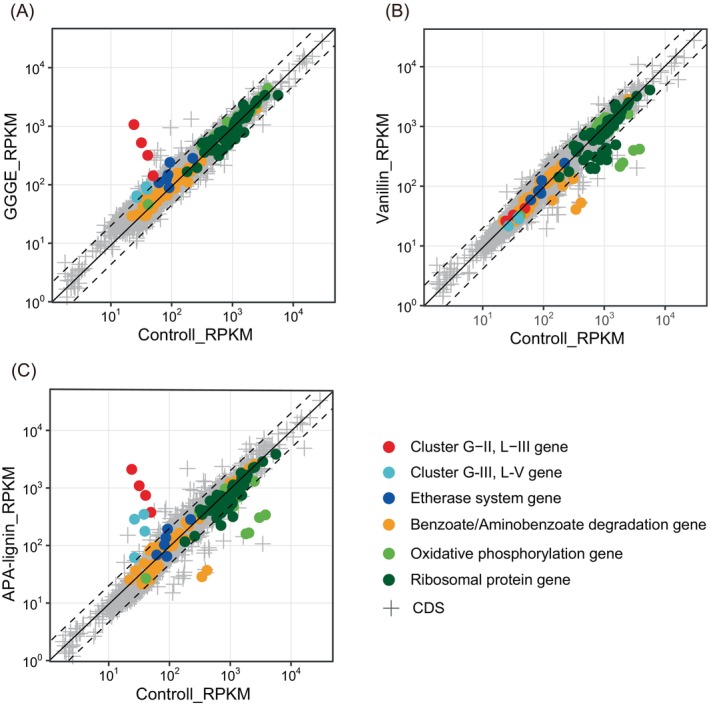
Comparison of global genomic expression in the presence of GGGE, vanillin and APA‐lignin. Red, light blue and dark blue circles represent expression values, RPKMs, of genes in clusters G‐II, L‐III and G‐III/L‐V, and genes of the previously identified etherase system, respectively. Orange, light green and dark green circles show expression values of genes involved in metabolic pathways for benzoate/aminobenzoate degradation, oxidative phosphorylation and ribosomal proteins, respectively. Grey “plus” symbols represent expression values of other genes (CDS) of strain MBES04 with expression (RPKM ≧ 1). The genes showing significant upregulation (2‐fold) and downregulation (0.5‐fold) are depicted above and below the dotted lines. (A) Expression values of GGGE treatments versus control. (B) Expression values of vanillin treatments versus control. (C) Expression values of APA‐treatments versus control. GGGE, guaiacylglycerol‐β‐guaiacyl ether; RPKM, mapped reads per length of transcript/1000 per total million reads.

**FIGURE 6:**
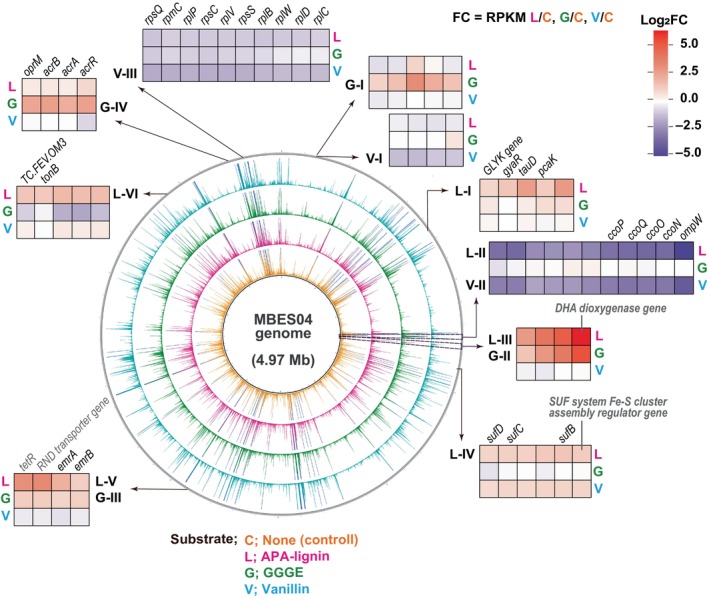
Overview of gene clusters of strain MBES04 differentially expressed in the presence of APA‐lignin, GGGE and vanillin. The expression level, RPKM, mapped to the MBES04 genome (grey ring) is represented as a line of colour depending on the tested substrate (None [control]; orange, APA‐lignin; magenta, GGGE; green, vanillin; light blue) within a threshold of 20,000 (Table S1A). The RPKMs over the threshold are indicated by blue lines. The distribution of differentially expressed gene clusters consisted of four or more genes (V‐I to V‐III, G‐I to G‐IV and L‐I to L‐IV) and are shown outside the rings. The heatmaps display the relative RPKMs to the control condition in the log_2_ function presenting the highest (red) and lowest (dark blue) induction. Possible functions based on the KO assignment and BLASTP search are labelled above the heatmap in black and grey, respectively. Each description of the assigned KO is described in Table S1B. Abbreviations are listed in Figure 5.

In the presence of 2.5 mM GGGE, a significant increase in the overall gene expression levels was observed. Specifically, the expression of 82 and 21 genes increased and decreased by >2‐ and 0.5‐fold, respectively (Figure 5A; Table S1A). Notably, the expression levels of three genes in clusters G‐I to G‐III (Figure 6; Table S1) were 2.2–44.6‐fold higher in the presence of GGGE than under control conditions (Figure 5A).

Cluster G‐I (gene loci MBENS4_0132 to MBENS4_0136) contains *pchF* and *pchC* genes; 4‐cresol dehydrogenase (hydroxylating) [EC:1.17.9.1 (transferred from EC:1.17.99.1)] flavoprotein and cytochrome subunits (Figure 6; Table S1), respectively. The enzyme plays a key role in the degradation of methoxylated aromatic compounds by oxidising the methyl group of the aromatic ring side chain in 4‐cresol and 2,4‐xylenol to a hydroxy moiety (Chen et al., 2014; Cunane et al., 2000). The formation of 4‐hydroxybenzoate from 4‐cresol through hydroxylation and subsequent oxidation by inducible enzymes enables further energy acquisition via the aromatic degradation pathway (Figure 1).

Of note, four genes in cluster G‐II (gene loci MBENS4_1158 to MBENS4_1161) were remarkably induced despite their low expression levels under control conditions (Figures 5A and 6), with expression changes of 2.87‐, 7.84‐, 16.46‐ and 44.58‐fold, respectively (Table S1). The most highly induced gene (MBENS4_1161) in the cluster G‐II is estimated as 2,4′‐dihydroxyacetophenone (DHA) dioxygenase, which produced 4‐hydroxybenzoate (C1 side chain) from DHA (C2 side chain) (Figure 1). The products of the other three genes remain hypothetical proteins. Further study is required at the protein level to understand the gene functions. A previous transcriptomics study of strain SYK‐6 (Varman et al., 2016) showed that GGGE repressed the overall expression levels of genes required for NAD(P)H production (except for genes that control the interconversion of NADH and NADPH), and strong induction of different genes was not reported.

Cluster G‐III encodes a transcriptional regulator, TetR and two sets of transporters of the resistance‐nodulation‐division (RND) family and the EmrAB complex. The RND family transporters are responsible for the efflux of various compounds, such as heavy metals, hydrophobic compounds, amphiphiles and nodulation factors in several bacteria such as strains of *Rizobium* (Alphaproteobacteria), *Ralstnia* (Betaproteobacteria) *Pseudomonas* (Gammaproteobacteria) and other genera (Nies, 2003; Zgurskaya et al., 2021). The EmrAB complex confers multidrug resistance and stable cellular stability in *E. coli* (Lomovskaya & Lewis, 1992). Cluster G‐IV encodes three multi‐drug efflux membrane proteins and a TetR/AcrR family transcriptional regulator involving multidrug resistance (Table S1B). Moreover, genes encoding the TetR family of transcriptional regulators were widely distributed in gram‐negative bacteria. Notably, the role of these genes in multidrug resistance has been demonstrated in *Salmonella* and other pathogenic bacteria (Colclough et al., 2019). However, further research is required to better understand the function of the TetR‐like regulators in Cluster G‐III and G‐IV of strain MBES04.

### Gene expression in the presence of vanillin

In some related bacteria, vanillin and vanillic acid are key intermediate metabolites directly involved in downstream pathways of various lignin‐related aromatic compounds, including various biaryls and monoaryls; GGGE, biphenyls, ferulic acid and etc (Bugg et al., 2011; Kamimura et al., 2017). In addition, vanillin and vanillic acid are major aromatic monomers nonbiologically produced from lignin by chemical oxidation (Ragauskas et al., 2014). Strain MBES04 has complete gene sets for vanillin assimilation via vanillic acid (Figure 1), which is consistent with previous results of our culture experiment using vanillin (1 mM) as a sole carbon source (Ohta et al., 2015). To confirm the effect of a lignin‐derived aromatic monomer, which is not a substrate for the etherase system, on gene expression, we analysed the gene expression profiles in the presence of vanillin.

To verify the viability of strain MBES04 cells in the presence of vanillin (2.5 mM), the difference in CFUs was assessed using the same method described above. In the presence of vanillin, the CFU showed an increase of 1.4 ± 0.2 × 10^8^ CFU/mL. The growth of the strain was within the same order of magnitude as that of the control (2.4 ± 0.1 × 10^8^ CFU/mL), despite partial inhibition. Additionally, the reaction of 2.5 mM vanillin with the cell suspension was analysed using LC–MS (Figure 4B). Vanillin was completely degraded within 2 h from the onset of exposure, indicating the high metabolic capability of the cells.

The presence of vanillin at 2.5 mM resulted in the overall suppression of gene expression, indicating toxicity to the strain. Specifically, the expression of 19 and 64 genes was upregulated and downregulated by >2‐ and 0.5‐fold, respectively (Figure 5B; Table S1). Notably, the expression levels of three genes in clusters V‐I to V‐III (Figure 6; Table S1) were 0.11–0.49‐fold lower in the presence of vanillin than under control conditions (Figure 5B; Table S1). Among the suppressed genes, severe suppression (0.11‐ to 0.13‐fold) was observed in the cluster V‐II (gene loci MBENS4_1124 to MBENS4_1133) (Figure 6) that includes genes for cytochrome *c* subunits (*ccoP*, *ccoQ*, *ccoO* and *ccoN*) in an oxidative phosphorylation complex compared to that in the control condition (Table S1). Additionally, the expression levels of the 30S and 50S ribosomal protein genes in cluster V‐III were greatly suppressed compared to thosein other conditions (gene loci MBENS4_0778 and MBENS4_0779, 025‐ and 0.28‐fold lower, respectively; MBENS4_4412 to MBES04g14421, 0.32‐ to 0.40‐fold lower) (Table S1; Figure 6).

In contrast, regarding genes associated with vanillin catabolism, two sets of out of three *ligAB* genes, which are aromatic ring‐opening dioxygenases, were significantly repressed (gene loci MBENS4_1991 and MBENS4_1992; 0.12‐ and 0.13‐fold, MBENS4_2242 and MBENS4_2243; 0.41‐ and 0.49‐fold lower expression, respectively). No significant changes in expression levels were observed for tandemly located *ligC* (gene locus 1g1953), and other *ligAB* genes (gene loci MBENS4_1954 and MBENS4_1955) (Table S1).

The *ligC* gene, which acts downstream of vanillin metabolism, and the NAD(P)^+^ transhydrogenase and phosphoglycerate kinase genes were strongly activated in the presence of vanillin, which is shown in a metabolomic study of strain SYK‐6 (Varman et al., 2016). This response was hypothesised to produce sufficient intracellular reducing power to support highly active gluconeogenesis. However, microarray analysis of strain SYK‐6 did not reveal detectable induction of the NAD transhydrogenase and phosphoglycerate kinase genes (Kamimura et al., 2017). Genes for aromatic ring and side chain processing enzymes were upregulated in the transcriptome analysis of *N. aromaticivorans* DSM12444 with a deletion of the gene *sacB* (SARO_RS09410, Saro_1879) supplemented with glucose and vanillin during its growth phase (Linz et al., 2021). These gene expression differences may be influenced by complex stress factors (Święciło & Zych‐Wężyk, 2013; Vargas‐Blanco & Shell, 2020) and growth phases (Orellana et al., 2017). Further research is required to fully understand the underlying mechanisms.

In this study, the severe repression of genes that play important roles in energy production (such as oxidative phosphorylation complex genes and *ligAB*) (Figure 1) indicated that energy production was suppressed with reduced production of ribosomal proteins in response to vanillin at a toxic concentration. Taken together with the rapid and complete degradation of vanillin, these results indicate that the strain entered a dormant state by the time of RNA preparation after catabolising and detoxifying vanillin.

### Gene expression in the presence of plant‐derived lignin

To compare the transcriptional responses to GGGE and vanillin with that to plant‐derived lignin, a lignin fraction, namely APA‐lignin (Nishimura et al., 2022), was used for the substrate. APA‐lignin is a lignin preparation holding the β‐O‐4 ether bond as a major intramolecular bond with minor contamination of plant polysaccharides and unidentified small lignin‐derived aromatic molecules. In this study, APA‐lignin was prepared from wood chips of *Eucalyptus globulus*. The primary constituents were the syringyl and guaiacyl units, with the syringyl unit being more prevalent. The *p*‐hydroxyphenyl unit was present in trace amounts. This was consistent with the earlier studies for the composition of E. globulus lignin (Nunes et al., 2010; Rencoret et al., 2011).

To verify the viability of strain MBES04 cells in the presence of 0.1% APA‐lignin, the difference of CFUs was assessed using the same method as above. The increase in CFU was 2.0 ± 0.2 × 10^8^ CFU/mL in the presence of APA‐lignin. The growth of strain occurred in the same order of magnitude as in the control (2.4 ± 0.1 × 10^8^ CFU/mL). To enhance our understanding of the microbial conversion of lignin fragments by strain MBES04, we analysed the reaction products from APA‐lignin by the cell suspension for 24 h under the same experimental conditions as those for the transcriptomic analysis using LC–MS (Figure 4C). By the time of RNA preparation, 4 h after exposure to APA‐lignin, GHP and syringylhydroxylpropanone (SHP) were produced at a detectable level. A dynamic change in the overall gene expression levels was observed. Specifically, the expression of 59 genes increased and decreased by >2‐ and 0.5‐fold, respectively (Figure 5C; Table S1). The overall profile of gene expression is likely a combination of differentially expressed genes in the presence of GGGE and vanillin. Severe suppression (0.03‐ to 0.21‐fold) was observed in cluster L‐II (gene loci MBENS4_1124 to MBENS4_1133) (Figure 6), which was the same gene set for cluster V‐II, compared to that under the control condition (Table S1). Cluster L‐III, the same gene set for cluster G‐II, showed tremendous expression (7.59‐ to 88.55‐fold) similar to the GGGE stimulus. Cluster L‐IV was upregulated by 2.11‐ to 2.79‐fold. Four out of six genes in the cluster were upregulated by vanillin as well. Cluster L‐IV contains three Fe‐S cluster assembly‐related proteins (SufD, SufC and SufB), a SUF system Fe‐S cluster assembly regulator, and two hypothetical proteins (Figure 6). Fe‐S cluster assembly proteins were known to be oxidative response proteins conserved in bacteria (Chiang & Schellhorn, 2012). The redox state and subunit structure of Fe–S clusters modulate environmental adaptation by regulating numerous biological processes, including respiration, photosynthesis, nitrogen fixation, DNA replication and repair, RNA modification and gene regulation (Mettert & Kiley, 2015). Cluster L‐V was detected at the same gene loci as cluster G‐III, which functions in detoxification by driving proton influx and drug efflux (Zgurskaya et al., 2021).

**FIGURE 7:**
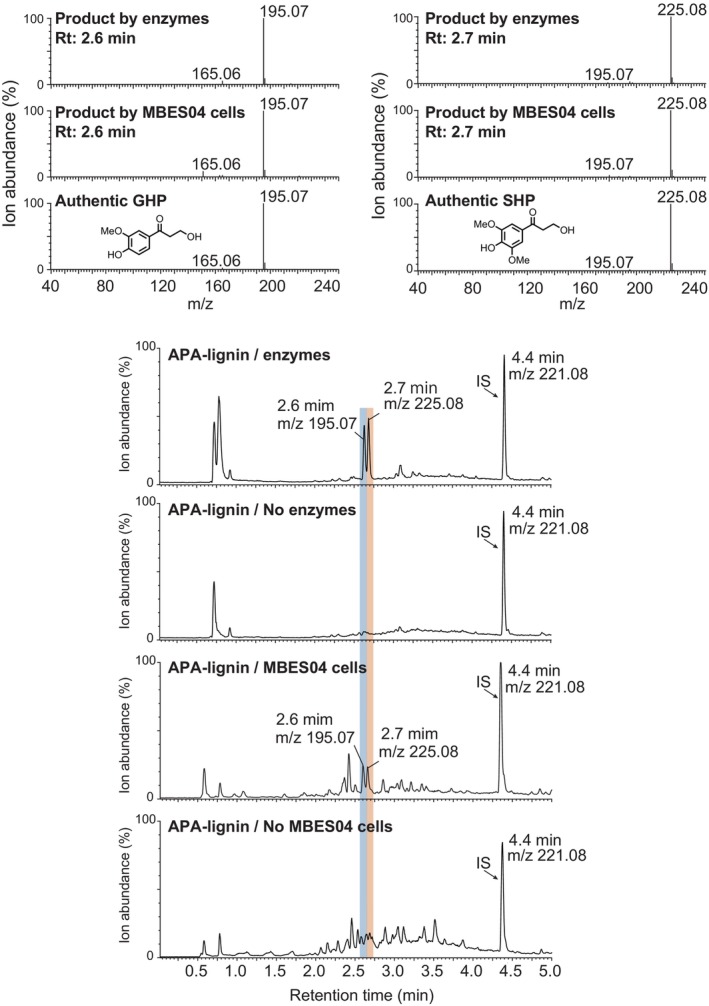
Total ion current chromatograms and mass spectra of enzyme and microbial reactions with APA‐lignin. Five enzymes (SDR3, SDR5 and GST3‐5) and 0.2% APA‐lignin were incubated with 20 mM reduced glutathione and 10 mM NAD sodium salt at pH 8.5 and 15°C for 24 h. Strain MBES04 was incubated in a basal medium containing 0.1% APA‐lignin at 30°C for 24 h. Samples were extracted by ethyl acetate and analysed by liquid chromatography‐mass spectrometry (LC–MS). IS, internal standard.

To obtain direct evidence of the involvement of the molecules produced during the degradation of APA‐lignin by the etherase system, we analysed the enzymatic reactions for APA‐lignin using LC–MS according to the method described in our previous report (Ohta et al., 2017) (Figure 7). The main reaction products generated by the etherase enzymes were GHP and SHP, consistent with those observed in strain MBES04 cells. Small amount of *p*‐hydroxyphenylhydroxylpropanone (HHP) was also detected in LC–MS extracted chromatograms based on the calculated monoisotopic mass of HHP. The peak areas of HHP under the chromatograms were 7.3 ± 0.6% by enzyme reactions and 5.4 ± 2.6% by MBES04 cells when those of GHP were considered as 100%. The results indicate that GGGE and β‐O‐4‐containing small molecules present in APA‐lignin are responsible for inducing changes in gene expression after being transported into the cells. Notably, GHP was a common reaction product generated from GGGE and APA‐lignin. This observation suggests the potential of GHP as an effector molecule responsible for the induction of common genes upon the addition of GGGE and APA‐lignin. Consequently, the etherase system confers a typical phenotypic feature to strain MBES04 when encountering the lignin fragments.

### Identification of effector molecules that induce the expression of cluster G‐II genes

**FIGURE 8:**
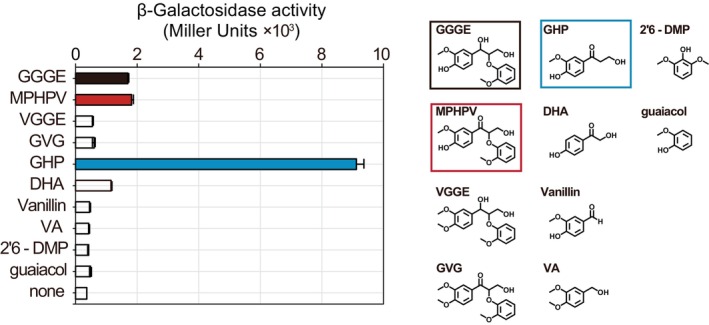
Screening of effector molecules that induce the expression of cluster G‐II genes. Gene expression activity from 600 bp of putative promoter of cluster G‐II (P_ClusterG‐II_) was assessed using a *lacZ*‐based promoter assay plasmid (pQF‐*lacZ*:: P_ClusterG‐II_). The β‐galactosidase activity of strain MBES04 holding pQF‐*lacZ*:: P_ClusterG‐II_, namely MBE*lacZ*, was measured after 16 h cultivation in basal medium supplemented with 1 mM each of lignin‐related compounds, GGGE, MPHPV, VGGE, GVG, GHP, DHA, vanillin, VA, 2,6‐DMP, or guaiacol, to screen for effector molecules. β‐Galactosidase activity was measured using *o*‐nitrophenyl β‐D‐galactopyranoside and expressed as Miller Units. The error bars represent the standard error of the mean of quadruplicate experiments. DHA, 2,4′‐dihydroxyacetophenone; DMP, dimethoxyphenol; GGGE, guaiacylglycerol‐β‐guaiacyl ether; GHP, guaiacylhydroxypropanone; GVG, β‐guaiacyl‐α‐veratrylglycerone; MPHPV, (2‐methoxyphenoxy) hydroxypropiovanillone; VA, veratryl alcohol; VGGE, veratrylglycerol‐β‐guaiacyl ether.

**FIGURE 9:**
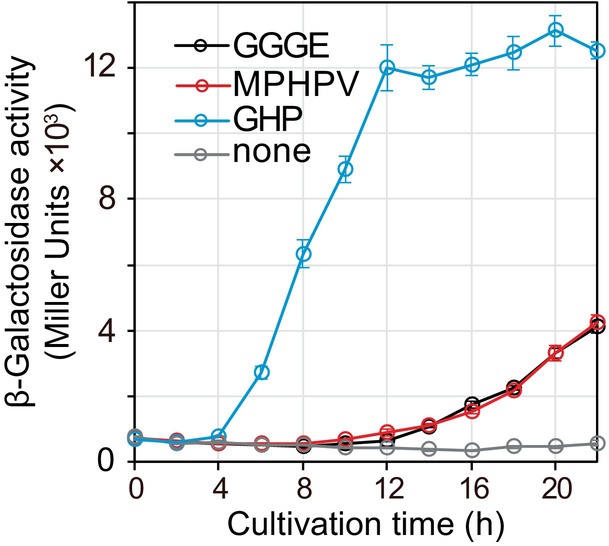
Determination of the effector molecule based on the time course of gene expression activity from P_ClusterG‐II_. Changes in β‐galactosidase activities of MBE*lacZ* cells cultured in basal medium supplemented with 1 mM GGGE, MPHPV, GHP, GGGE, MPHPV, or GHP were measured immediately after sampling every 2 h. Both GGGE and MPHPV were converted to GHP and guaiacol by the intrinsic etherase system of MBE*lacZ* during cultivation. β‐Galactosidase activity was assayed and expressed as Miller Units (see Figure 8). The error bars represent the standard error of the mean of quadruplicate experiments. Abbreviations are listed in Figure 8.

The transcription of clusters G‐II and L‐III initiated from the same starting point, which was estimated to be approximately 100 base pairs upstream of the cluster, based on the redundancy of RNA‐sequencing reads (Figure S1). We constructed a promoter assay plasmid (pQF‐*lacZ*:: P_ClusterG‐II_) by inserting the 1–600 bp upstream of MBENS4_1161 (the starting gene of cluster G‐II and L‐III, MBENS4_1158 to MBENS4_1161) directly upstream of the galactosidase gene start codon (Figure S2). Galactosidase activity of strain MBES04 transformed by pQF‐*lacZ*:: P_ClusterG‐II_, namely MBE*lacZ*, was measured in cultures supplemented with GGGE, VGGE, MPHPV, GVG, GHP, vanillin, VA, 2,6‐DMP, DHA, or guaiacol (Figure 8). The strongest induction was observed with GHP, and induction was also observed with GGGE, MPHPV and DHA. In contrast, no induction was observed with vanillin or methoxyphenol treatment. Our time‐course observations with GGGE, MPHPV and GHP showed that induction occurred earliest with GHP (after 6 h), whereas delayed induction was observed with GGGE and MPHPV (after 12 h), as shown in Figure 9. These results suggest that GGGE and MPHPV function as effector molecules after they are converted to GHP—the final reaction product from GGGE and MPHPV by the intrinsic etherase system of the strain. These results indicate that GHP acts as an effector molecule that induces the expression of genes in clusters G‐II and L‐III.

Effector molecules for genes related to the catabolism of lignin‐derived aromatic molecules have been reported for several bacteria that possess etherase systems (Kamimura et al., 2010, 2017; Uchendu et al., 2021). Vanillic acid, gallic acid, protocatechuic acid (Araki et al., 2019, 2020; Kamimura et al., 2017) and hydroxycinnamoyl‐CoAs (Kasai et al., 2012) are known to act as effectors of cis‐elements that regulate catabolism. A recent study of transcriptional regulation by the TetR‐type transcriptional repressor, PprR, in *Aromatoleum aromaticum* EbN1^T^ (classified as a Betaproteobacteria) indicated that environmental bacteria can sense very low concentrations of lignin‐derived 3‐phenylpropanoates by forming their coenzyme A thioesters as effectors (Vagts et al., 2021). This study demonstrated how bacteria regulate their metabolism to adapt to different nutritional conditions. To the best of our knowledge, this is the first study to show that phenylpropanone monomers act as effector molecules, but without promoting the degradation of phenylpropanone, they promote the degradation of other lignin derivative compounds.

### Metabolic implications of the etherase system

Degradation of phenylpropanone monomers generated from the etherase system requires activation of the C3 side chain with CoA and ATP, which is energetically demanding. Instead, strain MBES04 prefers to assimilate enzymatically degradable substances, such as glucose, DHA, vanillin, cresol, benzoate, with low energy cost. Such substrates are available in the environment because they are released via chemical and biological degradation of organic materials, such as plant biomass, especially from the lignin, and petroleum‐based chemicals.

At the case, we further considered what is the role of the etherase system for strain MBES04. As a sensing system for nutritional plant biomass, strain MBES04 constantly expresses genes for an etherase system that produces phenylpropanone from lignin fragments. From our comparative transcriptomic results, the phenylpropanone induced multiple enzymes, including dioxygenase for aromatic monomers with a C2 side chain and detoxifying transporters for a variety of recalcitrant toxic compounds. These metabolic traits are likely correlated with a survival strategy of the strain MBES04 to detect the presence of nutrients, and utilise more easily assimilated aromatic compounds rather than phenylpropanone, and also to gain the tolerance to the toxic aromatic compounds.

In order to clarify the metabolic traits, the cellular response to lignin fragments should be further investigated by means of proteomic and metabolic analyses or using living cells with knockouts of the respective genes. In the future, we will conduct further study on the functions of induced genes, more specifically, enzymatic properties of DHA dioxygenase and other enzymes, regulatory proteins, and binding domains in cluster G‐II/L‐III.

## AUTHOR CONTRIBUTIONS

**Eri Kumagawa:** Conceptualization (equal); funding acquisition (supporting); investigation (lead); methodology (equal); validation (equal); visualization (equal); writing – original draft (equal); writing – review and editing (equal). **Madoka Katsumata:** Conceptualization (supporting); investigation (equal); methodology (equal); writing – review and editing (supporting). **Hiroshi Nishimura:** Conceptualization (supporting); funding acquisition (equal); investigation (equal); methodology (equal); writing – review and editing (supporting). **Takashi Watanabe:** Conceptualization (equal); supervision (lead); validation (equal); writing – review and editing (equal). **Shun'ichi Ishii:** Conceptualization (supporting); investigation (equal); methodology (lead); validation (equal); visualization (equal); writing – review and editing (equal). **Yukari Ohta:** Conceptualization (lead); funding acquisition (lead); investigation (lead); project administration (lead); supervision (lead); writing – original draft (lead); writing – review and editing (lead).

## CONFLICT OF INTEREST STATEMENT

The authors declare no conflicts of interest associated with this manuscript.

## Supporting information


**Figure S1:** Close‐up view of RNA raw read mapping to Cluster G‐II/L‐III of MBES04 genome.
**Figure S2:** Map of the plasmid for promoter assay and nucleotide sequence of P_ClusterG‐II_.

**Table S1:** (A) Transcriptomic analysis data. (B) Clusters of differentially expressed genes in the presence GGGE, vanillin and APA‐lignin.
**Table S2:** Components of mineral medium.
**Table S3:** Primer sequences used in this study.
**Table S4:** Referenced enzymes in various degradation and metabolism pathway.

## Data Availability

The *Novosphingobium* strain MBES4 is deposited in the public culture collection of the National Institute of Technology Evaluation, Japan under accession number NBRC114556. The complete genome sequences and RNA sequence data were submitted to the DDBJ/EMBL/GenBank databases under accession numbers AP026899–AP026901 and DRR487002–DRR487005, respectively.
