# Supplementary material for: The etherase system of Novosphingobium sp. MBES04 functions as a sensor of lignin fragments through phenylpropanone production to induce specific transcriptional responses
Source: Environ Microbiol Rep. 2023 Nov 10;16(1):e13210. doi: 10.1111/1758-2229.13210 (PMC10866074; doi:10.1111/1758-2229.13210)
Supplement: Supplementary file 1 — Figure S1: Close‐up view of RNA raw read mapping to Cluster G‐II/L‐III of MBES04 genome. Figure S2: Map of the plasmid for promoter assay and nucleotide sequence of PClusterG‐II. [file EMI4-16-e13210-s002.pdf]

Supplementary figures

The etherase system of *Novosphingobium* sp. MBES04 functions as a sensor of lignin fragments through phenylpropanone production to induce specific transcriptional responses

Running title: Role of the *Novosphingobium* etherase system

Eri Kumagawa[1], Madoka Katsumata[1], Hiroshi Nishimura[2], Takashi Watanabe[2], Shun'ichi Ishii[3]*, Yukari Ohta[1]*

[1] Gunma University Center for Food Science and Wellness, 4-2 Aramaki, Maebashi, Gunma University, Gunma, 371-8510, Japan
[2] Research Institute for Sustainable Humanosphere, Kyoto University, Gokasho, Uji City, Kyoto Prefecture, 611-0011, Japan
[3] Institute for Extra-cutting-edge Science and Technology Avant-garde Research (X-star), Japan Agency for Marine-Earth Science and Technology (JAMSTEC), 2-15 Natsushima, Yokosuka, Kanagawa, 237-0061, Japan

Co-author email addresses:
Eri Kumagawa; t212a001@gunma-u.ac.jp
Madoka Katsumata; m31001548@gmail.com
Hiroshi Nishimura; hiroshi_nishimura@rish.kyoto-u.ac.jp
Takashi Watanabe; watanabe.takashi.3a@kyoto-u.ac.jp
Shun'ichi Ishii; sishii@jamstec.go.jp

*Corresponding authors:
Yukari Ohta; Gunma University Center for Food Science and Wellness, Gunma University, 4-2 Aramaki, Maebashi, Gunma 371-8510, Japan; Email: y-ohta@gunma-u.ac.jp
ORCID: 0000-0001-5645-2311

Shun'ichi Ishii; Institute for Extra-cutting-edge Science and Technology Avant-garde Research (X-star), Japan Agency for Marine-Earth Science and Technology (JAMSTEC), 2-15 Natsushima, Yokosuka, Kanagawa, 237-0061, Japan, Email: sishii@jamstec.go.jp
ORCID: 0000-0002-0203-8569

Figure S1. Close-up view of RNA raw read mapping to Cluster G-II / L-III of MBES04 genome.

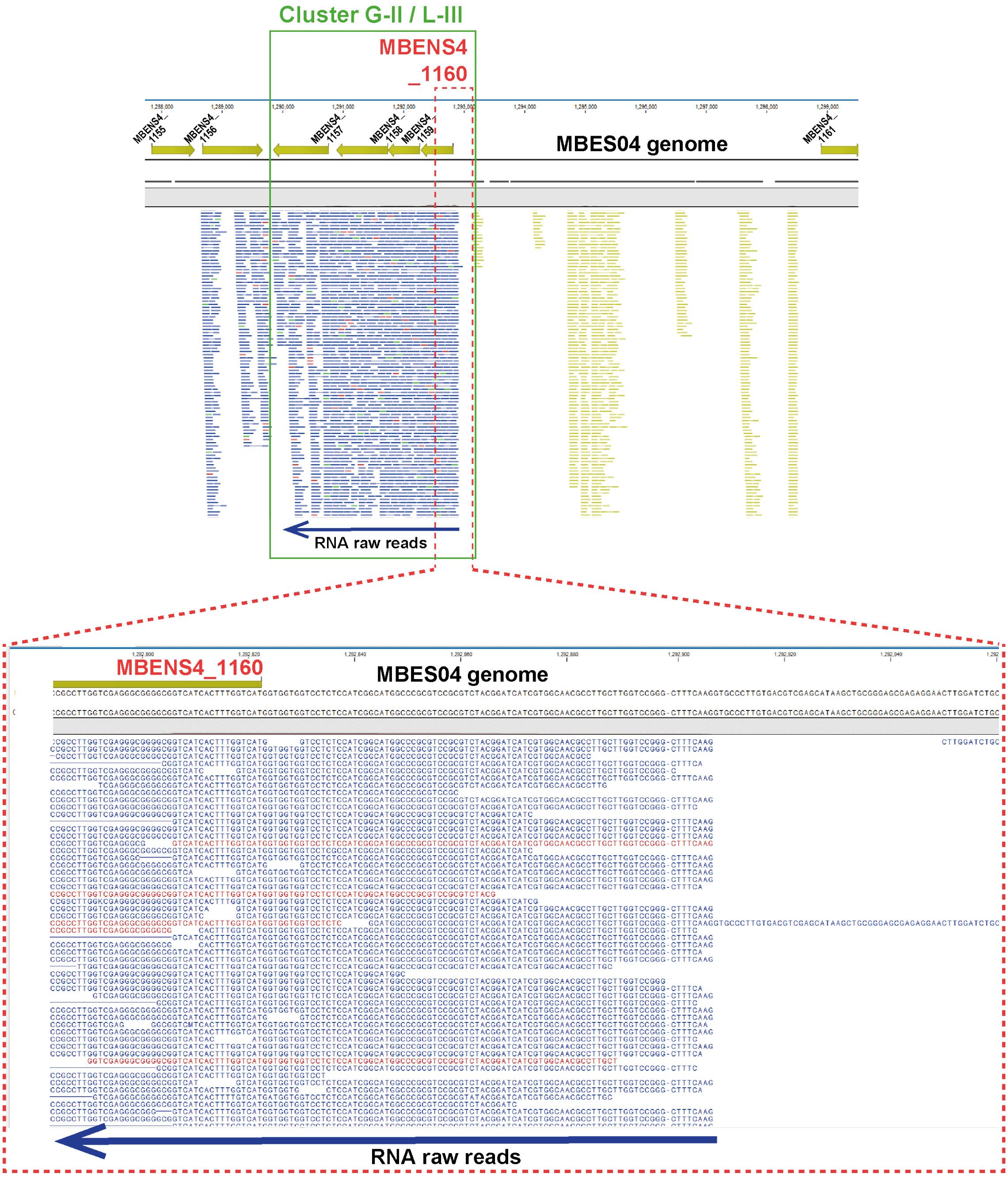

Figure S2. Map of the plasmid for promoter assay and nucleotide sequence of $P_{ClusterG\text{-}II}$

(a) Map of the plasmid for promoter assay

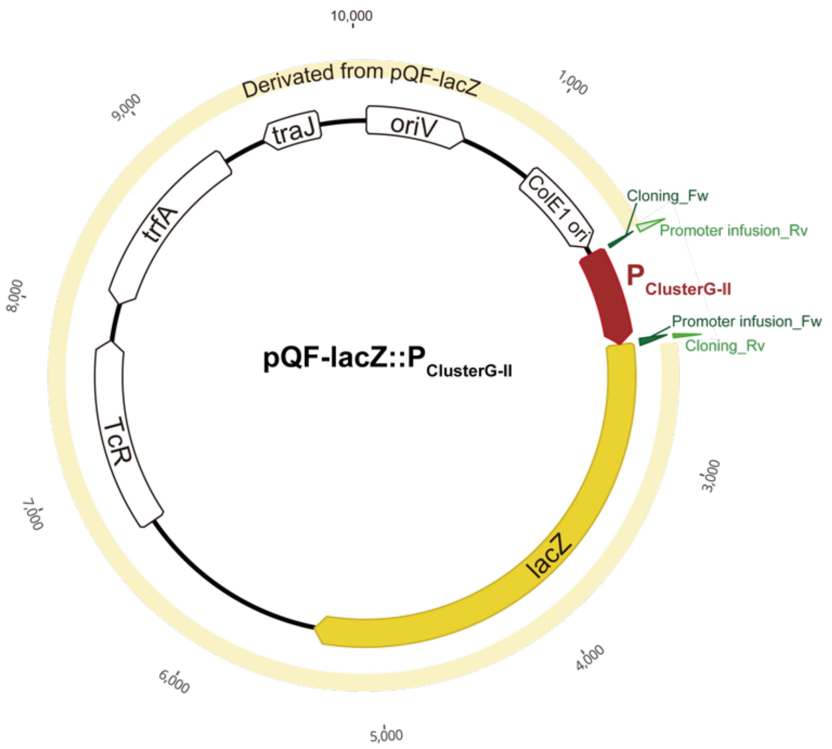

Features of pQF-lacZ::$P_{ClusterG\text{-}II}$

Label	Location	Note
oriV	11..642	incP origin of replication
ColE1 ori	1105..1693	high-copy-number ColE1/pMB1/pBR322/pUC origin of replication
$P_{ClusterG\text{-}II}$	1738..2337	600 bp region including a putative promoter for Cluster G-II and L-III
lacZ	2338..5412	β-galactosidase gene
TcR	6659..7858	tetracycline efflux protein gene (*tetA*)
trfA	8089..9237	trans-acting replication protein (oriV activator) gene
traJ	9509..9880	oriT-recognizing protein gene (*traJ*)

(b) Nucleotide sequence of $P_{ClusterG\text{-}II}$

><u>CCCATCTCGAACTCGGACG</u>TGAAACCTGCCAGCGCCGATGGTACTTACGCTCAAGCGT
TGGAAGAGTAGGACGTCGCCAGGCATTGTAGCCAGCGCAAGATCATAAAAACCCATCA
CAATGACAAAGGACCTCAATGGTCCACAAAGGGGGCCCAAAAGCCCCCTTTTTGCGTCT
CTCAAATGAACGCTCAACATCGGTGACGCGGGGTGGAGCAGCCCGCCGTTCAGATCGA
CAACGTCGAGATGAACATCAAATATCTGCTAACGGGCCTTAAACGGTCCATCATGGTGA
CGCGGGGTGGAGCAGCCCGGTAGCTCGTCAGGCTCATAACCTGAAGGTCGTAGGTTCA
AATCCTACCCCCGCAACCAACTATAACACTCGCCAAGCCCCCCGCCGCTCCTGCGCCGG
GGGGCTTGGCGTATCTGGGACACGCGTGCGCGCCTGTCCCGATCGCGCTGCGCGCAGAT
CCAAGTTCCTCTCGCTCCCGCAGCTTATGCTCGACGTCACAAGGGCACCTTGAAAGCCC
GGACCAAGCAAGGCGTTGCCACGATGATCCGTAGACGCGGACGCGGGCCATGCC<u>GATG</u>
<u>GAGAGGACCACCACC</u>

*Sequence corresponding to cloning primers (PClusterG-II_ Cloning Fwd and Rev, Supplementary TableS3) are underlined.
